# MSAdaNet: An Adaptive Multi-Scale Network for Surface Defect Detection of Smartphone Components

**DOI:** 10.3390/s26072091

**Published:** 2026-03-27

**Authors:** Jianqing Wu, Hong Chen, Xiangchun Yu, Shuxin Yang, Weidong Huang, Fei Xie, Hanlin Hong, Hui Wang

**Affiliations:** 1 School of Information Engineering, Jiangxi University of Science and Technology, Ganzhou 341000, China; jianqing.wu@jxust.edu.cn (J.W.); 6720230793@mail.jxust.edu.cn (H.C.); yangshuxin@jxust.edu.cn (S.Y.); 9119900008@jxust.edu.cn (W.H.); 9320090328@jxust.edu.cn (H.W.); 2 School of Information Engineering, Wenzhou Business College, Wenzhou 325000, China; 20249103@wzbc.edu.cn (F.X.); 23210816050@wzbc.edu.cn (H.H.)

**Keywords:** smartphone components inspection, defect detection, multi-scale adaptive network, object detection, YOLO

## Abstract

The detection of surface defects on smartphone components is a critical step in quality assurance for industrial manufacturing. However, existing deep learning-based methods struggle with the extreme variations in defect morphology and scale, while labeled training data remains scarce due to the high cost of expert annotation. To address these challenges, we propose a twofold solution. First, we introduce MSAdaNet, a Multi-Scale Adaptive Defect Detection Network, which integrates three novel modules: a Parallel Multi-Scale Feature Aggregation (PMSFA) backbone, a Focusing Diffusion Pyramid Network (FDPN) neck, and a Scale-Adaptive Shared Detection (SASD) head. Second, to combat data scarcity, we propose a novel data generation pipeline, creating the synthetic Smartphone Camera Bezel Dataset (SCBD) of 4936 images. Extensive experiments on both real-world and synthetic datasets validate our approach. On the challenging public SSGD, MSAdaNet achieves a state-of-the-art mAP@0.5 of 54.8%, outperforming prominent frameworks and improving upon the strong YOLOv11m baseline by +10.6 points in mAP@0.5 and +18.3 points in recall. Furthermore, on our synthetic SCBD, the model achieves an impressive 94.0% mAP@0.5, confirming the quality of our data generation pipeline and the robustness of our architecture across different data distributions. Ablation studies systematically confirm the significant contribution of each proposed module, validating MSAdaNet as an effective and efficient solution for industrial defect detection.

## 1. Introduction

With the proliferation of edge-to-edge displays and multi-camera modules in modern smartphones, the quality control of optical components such as screen glass and camera bezels has become critically important. These glass components are highly susceptible to defects during manufacturing and assembly, including micro-scratches, structural cracks, contamination spots, and coating damage. Such defects not only degrade the surface quality of the product but also directly impair its functional integrity. Consequently, industrial quality inspection standards for smartphone glass components have become increasingly stringent, necessitating high-precision automated defect detection systems. Traditional non-destructive testing methods, like manual visual inspection, are no longer adequate for modern manufacturing demands due to their low efficiency, high labor costs, and poor consistency [1]. Therefore, developing an automated defect detection system using machine vision technology to improve accuracy and efficiency has emerged as a crucial research direction in this field.

Defect detection methods for smartphone components can be broadly classified into two main categories. The first category comprises traditional machine vision methods, which rely on manually designed feature extractors tailored for specific detection tasks [2]. However, due to the limited expressive power of these handcrafted features, their detection performance, stability, and generalization capability are often unsatisfactory when confronted with the diverse morphologies of component defects [3]. The other major category consists of deep learning-based methods. Benefiting from their powerful ability to extract high-level abstract features, these methods generally achieve superior detection performance. In recent research, object detection frameworks have been widely employed for the classification and localization of component defects. In one representative study, Han et al. constructed a dataset for smartphone screen defects and experimentally validated the applicability of several classical object detection frameworks, such as Faster R-CNN, ATSS, and YOLOX [4,5,6,7]. Their results indicate that these frameworks provide a viable technical route for detecting defects on smartphone components.

**Figure 1:**
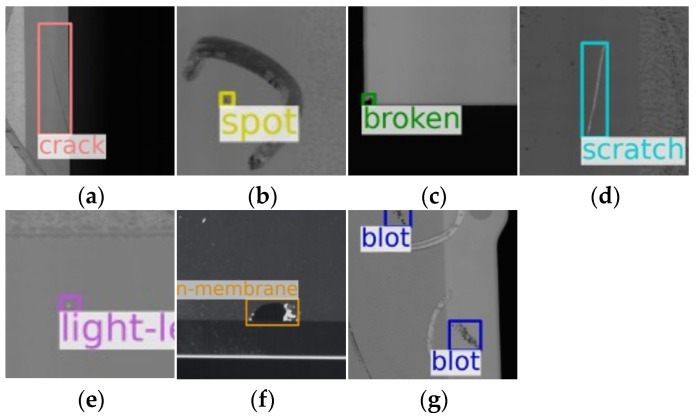
Seven types of defects in Smartphone Screen Glass Dataset (SSGD): (**a**) Crack, (**b**) Spot, (**c**) Broken, (**d**) Scratch, (**e**) Light-Leakage, (**f**) Broken-Membrane, (**g**) Blot.

However, when generic object detection models are directly applied to this task, their performance remains insufficient, largely due to fundamental differences between industrial defect detection and natural image analysis. As illustrated in Figure 1, this task is impeded by three primary challenges. First, unlike natural objects, defects exhibit extreme inter- and intra-class morphological variation, which complicates robust feature extraction. Second, defects span a vast range of scales, from micro-defects occupying only a few pixels to large structural failures that can exceed the limited receptive fields of standard models, necessitating advanced multi-scale feature representation. Finally, the field suffers from severe data scarcity due to the prohibitive cost of manual annotation and the privacy-sensitive nature of industrial data. This bottleneck severely impedes the ability of a model to learn diverse defect patterns and generalize to real-world manufacturing scenarios.

To combat these deficiencies, recent research has explored several avenues. While some approaches have incorporated attention mechanisms to improve adaptability, these modules are primarily optimized for salient objects in natural images and thus yield only marginal improvements for the subtle features of industrial defects [8,9,10,11]. Other methods have explored multi-scale feature fusion schemes to handle varying defect sizes; however, their reliance on high-resolution feature maps and global attention modules introduces substantial computational overhead, compromising real-time performance [12,13,14]. In particular, architectures such as FPN and PAN rely on sequential top-down or bottom-up pathways. Consequently, they lack direct interaction between non-adjacent feature levels, which results in accumulated semantic loss when handling the extreme scale differences characteristic of smartphone component defects. Furthermore, these studies are often constrained to a limited set of defect classes, failing to enhance overall system robustness. Therefore, a detection framework that is both highly accurate and efficient, explicitly designed for the unique characteristics of smartphone component defects, is urgently needed.

To address this gap, this paper proposes the Adaptive Multi-Scale Network (MSAdaNet). Our framework integrates a novel backbone and detection head with a Focusing Diffusion Pyramid Network (FDPN) neck [15] to enhance feature extraction for defects of varying shapes and sizes while maintaining real-time inference efficiency. Our primary contributions are as follows:

(1) We design a novel Parallel Multi-Scale Feature Aggregation (PMSFA) backbone that employs parallel convolutional kernels of varying sizes. This allows the network to effectively capture defect details across multiple scales simultaneously, demonstrating significant advantages in detecting minute defects.
(2) We introduce a Scale-Adaptive Shared Detection (SASD) head that improves accuracy and localization through cross-task parameter sharing and dynamic, scale-adaptive feature transformations that adjust feature representation according to defect scale.
(3) We develop a comprehensive data enrichment strategy to address data scarcity, combining synthetic data generation with a systematic augmentation pipeline to robustly expand the training dataset.

## 2. Related Works

### 2.1. Defect Detection on Smartphone Components

Early research on smartphone component defects primarily focused on image classification, leveraging traditional machine vision techniques. For instance, some studies employed multifractal spectral features with Support Vector Machines (SVMs) for classification, while others utilized texture features like Local Binary Patterns (LBP) and Gray Level Co-occurrence Matrices (GLCM) to identify screen irregularities [16,17]. The advent of deep learning saw the application of early Convolutional Neural Networks (CNNs) for classification-only tasks [18]. However, these methods were limited as they could only identify if an image contained a defect, without providing any localization information, and struggled with minute or inconspicuous defect types. More recently, the focus has shifted towards object detection frameworks to achieve both classification and localization. Mao et al. enhanced a YOLOv5-based detector with multi-scale feature extraction and attention modules to improve accuracy [19]. Similarly, Wu et al. improved feature representation using multi-scale fusion and hybrid attention mechanisms, achieving a mAP of 62.46% on an industrial dataset [20]. Despite these advances, existing methods often adapt techniques designed for natural images, which do not fully capture the unique, complex morphologies of industrial defects, thus limiting their performance and robustness across diverse defect types.

### 2.2. Object Detection Architectures

Modern object detection is largely categorized into one-stage detectors and two-stage (e.g., Fast R-CNN [21], Faster R-CNN). One-stage detectors, exemplified by the YOLO series [22,23,24,25,26,27] and ATSS, have achieved real-time performance by eliminating the region proposal step, making them highly suitable for industrial applications. A state-of-the-art model like YOLOv11 typically features architectural advancements over its predecessors, such as modified C2f blocks, attention-like layers, and specialized convolutions in the detection head, while continuing to use loss functions like CIoU for bounding box regression. Concurrently, Transformer-based architectures [28,29,30] have demonstrated exceptional performance on large-scale datasets through their self-attention mechanisms, representing another significant direction in the field. However, their self-attention mechanisms incur quadratic computational costs with feature map resolution and typically require large-scale pre-training. These characteristics make them less suited to data-scarce, real-time industrial inspection scenarios.

### 2.3. Small Object Detection

The detection of small objects is a persistent challenge in computer vision, primarily due to the sparse feature information available in low-resolution targets. Two principal strategies have been developed to address this. The first, multi-scale feature representation, aims to fuse the detailed positional information from low-level feature maps with the rich semantic information from high-level maps [31]. Seminal works in this area include the Feature Pyramid Network (FPN) and its derivatives like the Path Aggregation Network (PAN) [32,33]. Other approaches leverage attention mechanisms such as Squeeze-and-Excitation Networks, Selective Kernel Networks or Split-Attention Networks to dynamically weigh features from different scales. The second strategy involves leveraging contextual information to enrich the features of a small object by incorporating information from its surrounding environment, often by using a larger detection window or by modeling local, semantic, or spatial context [34,35,36,37,38,39].

## 3. The Proposed Method

**Figure 2:**
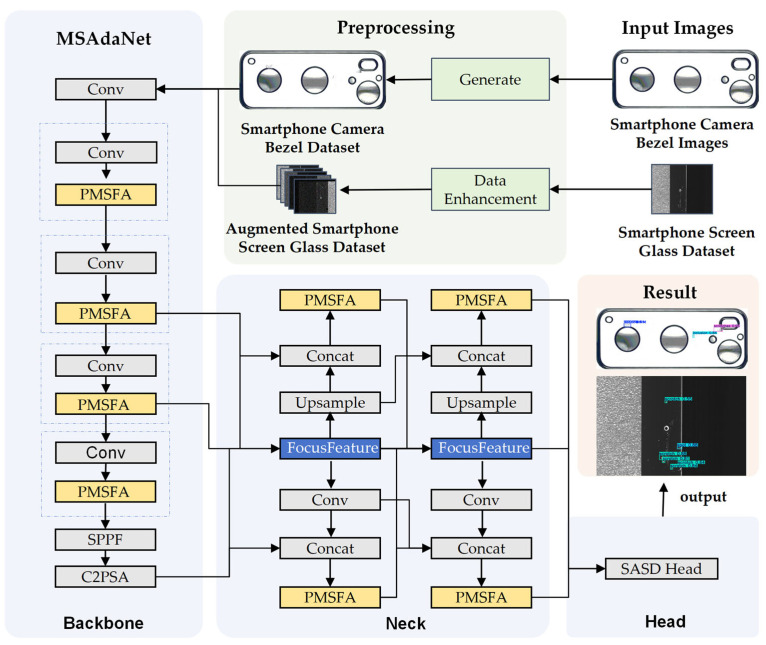
Overview of the proposed MSAdaNet framework.

The proposed MSAdaNet framework, illustrated in Figure 2, is designed to address the critical challenge of insufficient and diverse defect samples in the industrial inspection of smartphone components. The framework introduces a novel data synthesis and augmentation pipeline to train a robust deep learning model for accurately identifying defects on both smartphone camera bezels and screen glass.

### 3.1. Overall Framework of MSAdaNet

The core of this framework is the MSAdaNet, a single-stage object detection architecture designed to automatically detect and localize defects. As illustrated in Figure 2, the network is composed of three primary modules: a backbone for feature extraction, a neck for multi-scale feature aggregation, and a head for final prediction.

(1) The backbone network processes input images to extract hierarchical feature maps. We introduce a novel PMSFA module as the core of our backbone. By employing parallel convolutional kernels of varying sizes, the PMSFA is designed to efficiently capture defect details across multiple scales, which is particularly advantageous for detecting minute defects. Residual connections are incorporated into deeper layers to ensure robust feature flow. The depth and width of the backbone are designed to balance accuracy and efficiency, consistent with principles from the YOLO series. A detailed description of the PMSFA module is provided in Section 3.2.
(2) Feature maps extracted from multiple stages of the backbone are fed into the neck network for multi-scale feature fusion. We utilize the FDPN as the neck module to effectively aggregate features from different scales while maintaining high computational efficiency. Further details are presented in Section 3.3.
(3) The aggregated feature maps from the neck are then passed to the detection head for final class and bounding box prediction. We propose a SASD head, which introduces two key mechanisms to enhance detection accuracy. First, it employs cross-task parameter sharing between the classification and localization branches, enabling mutual feature learning. Second, it incorporates scale-adaptive feature transformations, which use learnable scale factors to dynamically adjust feature representations based on defect scale. This design significantly improves the ability of the model to accurately localize defects, especially in challenging scenarios involving complex scale distributions. The SASD head is detailed further in Section 3.4.

### 3.2. Parallel Multi-Scale Feature Aggregation Module

The C3K2 module, the core feature extractor in the YOLOv11 backbone, is sub-optimal for the specific challenges of smartphone component defect detection. Its fixed receptive field struggles with the inherently multi-scale nature of defects, which can range from pixel-level bright spots to linear scratches. Furthermore, its standard bottleneck structure can lead to the loss of fine-grained details, increasing the rate of missed detections for small defects. While existing multi-scale architectures offer partial solutions, Inception incurs substantial computational redundancy by applying standard convolutions across all channels in every parallel branch [40], and the cascaded inter-subset information flow of Res2Net risks diluting fine-grained micro-defect features through inter-scale mixing [41].

**Figure 3:**
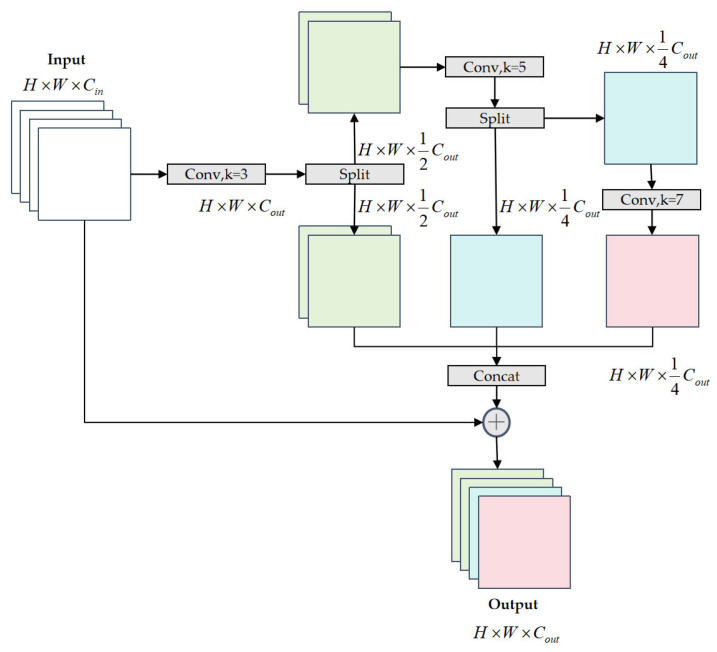
The structure of the PMSFA module. Different colors are used to visually distinguish feature maps at different stages.

To address these limitations, we propose the PMSFA module. As illustrated in Figure 3, this module replaces the C3K2 block in the backbone to enhance multi-scale feature representation while maintaining computational efficiency. The design is based on a “divide-and-conquer” strategy, using a parallel branching architecture to avoid the information attenuation common in deep serial structures.

Given an input feature map $X\in R^{H\times W\times C_{in}}$, the PMSFA module first applies a 3 × 3 standard convolution for preliminary feature enhancement:

(1) $$\begin{array}{c} F_{0}=\sigma (BN\left({Conv}_{3\times 3}\left(X\right)\right))\in R^{H\times W\times C_{out}} \end{array}$$

where $\begin{array}{c} \sigma (\cdot ) \end{array}$ denotes the SiLU activation function and $\begin{array}{c} BN(\cdot ) \end{array}$ represents the batch normalization operation.

Next, the module employs a progressive channel partitioning strategy. The enhanced feature map $\begin{array}{c} F_{0} \end{array}$ is bisected along the channel dimension into $F_{0}^{\left(1\right)}$ and $F_{0}^{\left(2\right)}$. The first branch $F_{0}^{\left(1\right)}$ is processed by a 5 × 5 depthwise separable convolution to extract medium-scale features:

(2) $$\begin{array}{c} F_{1}=\sigma (BN({DWConv}_{5\times 5}(F_{0}^{\left(1\right)})))\in R^{H\times W\times \frac{1}{2}C_{out}} \end{array}$$

where ${DWConv}_{5\times 5}(\cdot )$ represents a depthwise convolution. This new feature map $F_{1}$ is again bisected into $F_{1}^{\left(1\right)}$ and $F_{1}^{\left(2\right)}$. The first half, $F_{1}^{\left(1\right)}$, is then processed by a 7 × 7 depthwise convolution to capture large receptive field features:

(3) $$\begin{array}{c} F_{2}=\sigma (BN({DWConv}_{7\times 7}(F_{1}^{\left(1\right)})))\in R^{H\times W\times \frac{1}{4}C_{out}} \end{array}$$

This parallel, non-overlapping processing allows for the extraction of a diverse set of features without redundancy. Following this, the features from all three parallel paths, representing small, medium, and large receptive fields respectively, are aggregated via channel concatenation:

(4) $$\begin{array}{c} F_{concat}=Concat(F_{2},F_{1}^{\left(2\right)},F_{0}^{\left(2\right)})\in R^{H\times W\times C_{out}} \end{array}$$

Finally, a 1 × 1 convolution fuses the concatenated features, while a residual connection from the original input is added to preserve information and enhance gradient flow:

(5) $$\begin{array}{c} F_{out}={Conv}_{1\times 1}(F_{concat})+X \end{array}$$

By replacing the standard C3K2 module with our PMSFA module, the backbone can construct a rich, hierarchical feature representation better suited to the diverse scales of smartphone defects, while the use of depthwise separable convolutions ensures high computational efficiency.

### 3.3. Focusing Diffusion Pyramid Network

**Figure 4:**
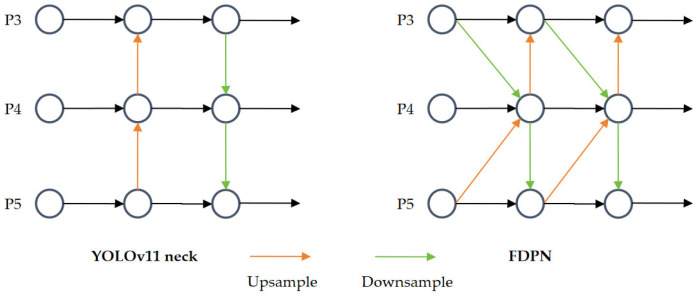
The difference between YOLOv11 neck and FDPN.

Standard feature pyramid architectures like FPN and PAN, while foundational, suffer from several limitations in the context of defect detection. Their sequential pathways create large semantic gaps between non-adjacent feature levels, and their uniform fusion operations fail to adaptively select the most discriminative information from each scale. To overcome these limitations, we use a novel neck architecture, the FDPN. The key innovation of this architecture lies in its core building block, the FocusFeature module, which performs direct, parallel fusion of non-adjacent feature levels. As shown in Figure 4, the FDPN architecture is designed for more direct and efficient multi-scale information fusion compared to the PAN structure used in YOLOv11.

**Figure 5:**
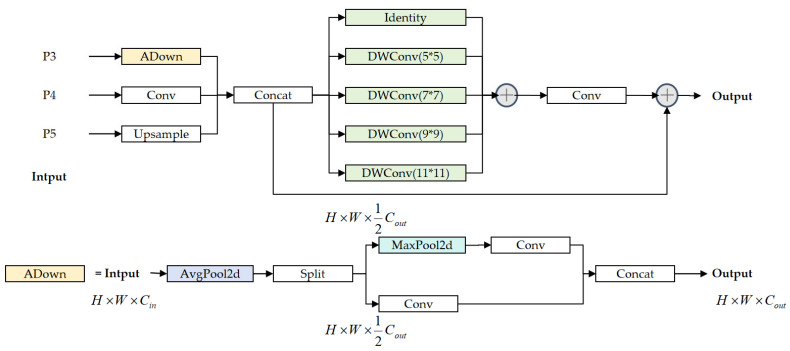
The structure of the FocusFeature module.

The core of FDPN is the FocusFeature module, illustrated in Figure 5. It processes inputs from three levels P3/8, P4/16, and P5/32 through two sequential stages: scale alignment and multi-scale fusion with refinement.

The first stage, scale alignment, transforms the input features to a common resolution and channel dimension. The high-resolution feature is downsampled using the ADown module. This module first applies average pooling, then splits the result channel-wise into parallel spatial and channel branches. The spatial branch uses a 3 × 3 convolution to capture structural information, while the channel branch employs max pooling and a 1 × 1 convolution to preserve salient features. The outputs are then concatenated, effectively retaining both spatial structure and key channel-wise information. This operation can be summarized as:

(6) $$F_{avg}={AvgPool}_{2\times 2}^{s=1}\left(P_{3}\right)$$

(7) $$F_{avg1},F_{avg2}=Split(F_{avg})$$

(8) $$F_{spatial}={Conv}_{3\times 3}^{s=2}(F_{avg1})$$

(9) $$F_{channel}={Conv}_{1\times 1}({MaxPool}_{3\times 3}^{s=2}(F_{avg2}))$$

(10) $$F_{ADown}=Concat(F_{spatial},F_{channel})$$

The intermediate-scale feature undergoes a lightweight 1 × 1 convolution for channel compression:

(11) $$\begin{array}{c} F_{mid}=Conv_{1\times 1}(P_{4}) \end{array}$$

The low-resolution feature is brought to the target resolution via a 1 × 1 convolution followed by 2× bilinear upsampling:

(12) $$\begin{array}{c} F_{up}=Conv_{1\times 1}({Upsample}_{2\times }(P_{5})) \end{array}$$

Once aligned, the three feature streams $F_{ADown}$, $F_{mid}$, and $F_{up}$ are concatenated into a single feature map $F_{fused}$. This unified representation is then processed through a residual block designed for multi-scale feature enhancement. First, parallel depthwise convolutions with multiple kernel sizes *K* = {5, 7, 9, 11} are applied to capture defect patterns of various sizes, and the results are refined with a pointwise convolution:

(13) $$F_{enhanced}=F_{fused}+\sum_{k\epsilon K}{DWConv}_{k\times k}(F_{fused})$$

(14) $$\begin{array}{c} F_{focused}=PWConv(F_{enhanced}) \end{array}$$

A residual connection is then added to combine the refined features with the original concatenated map, facilitating gradient flow and enabling the learning of fine-grained variations:

(15) $$\begin{array}{c} F_{out}=F_{fused}+F_{focused} \end{array}$$

This architectural design offers several key advantages. First, the direct fusion of non-adjacent pyramid levels reduces information loss and computational cost. Second, the multi-kernel depthwise convolution provides adaptive receptive fields to capture defects across a wide scale range, from pixel-level anomalies to large structural defects. Finally, the dual-pooling operation of the ADown module effectively retains both detailed texture and semantic information during the downsampling process.

### 3.4. Scale-Adaptive Shared Detection Head

The standard YOLOv11 detection head suffers from two primary drawbacks for smartphone component defect detection. First, its independent branches process multi-scale features in isolation, making them scale-agnostic and unable to adapt to the fact that small defects require fine spatial detail while large defects demand high-level semantic context. Second, the complete separation of classification and localization tasks prevents collaborative feature learning between these two closely related objectives.

**Figure 6:**
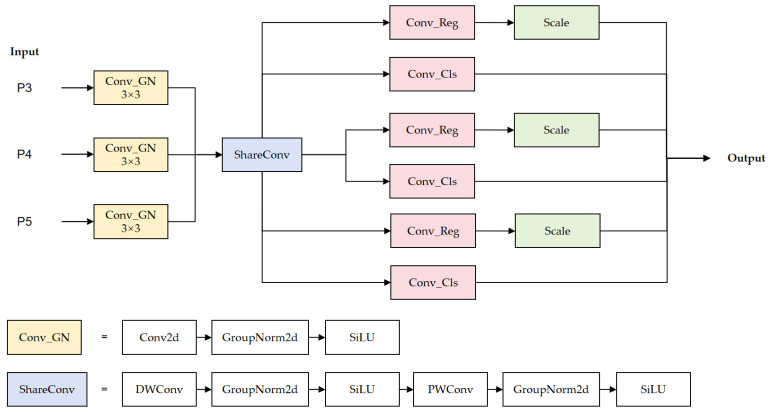
The structure of the SASD head.

To address these issues, we propose the SASD, which introduces cross-task parameter sharing and a scale-adaptive regression mechanism. Inspired by the per-level trainable scalars in FCOS for scale-aware regression [42], SASD extends this idea by applying learnable scale factors exclusively to the regression branch within a GroupNorm-based shared convolutional architecture. This enables precise regression calibration at each detection scale while maintaining parameter efficiency. The architecture, detailed in Figure 6, first creates a unified feature space and then performs decoupled predictions. Given the multi-scale feature maps $\left\{F_{i}\right\}$ from the FDPN neck, the SASD head first projects them into a common hidden feature space via a scale-adaptive convolutional block:

(16) $$H_{i}=\sigma (GN({Conv}_{3\times 3}^{\left(i\right)}(F_{i})))$$

where ${Conv}_{3\times 3}^{\left(i\right)}$ is the layer-specific convolution, followed by Group Normalization (GN), and σ is the activation function.

Subsequently, a parameter-efficient shared module performs deep feature extraction:

(17) $$\begin{array}{c} H_{i}^{\prime }=PWConv(DWConv(H_{i})) \end{array}$$

where DWConv and PWConv denote depthwise and pointwise convolutions, respectively.

Specifically, SASD introduces learnable, layer-wise scale factors $a_{i}$ to adaptively modulate the bounding box regression branch for different feature semantics:

(18) $$\begin{array}{c} {Reg}_{i}=a_{i}\cdot {Conv}_{3\times 3}^{\left(reg\right)}(H_{i}^{\prime }) \end{array}$$

(19) $$\begin{array}{c} {Cls}_{i}={Conv}_{3\times 3}^{\left(cls\right)}(H_{i}^{\prime }) \end{array}$$

For each feature level *i*, the regression branch outputs a tensor of shape (4 × (*L* + 1)) for Distribution Focal Loss (DFL) based regression, while the classification branch outputs a tensor of shape C representing class scores. ${Reg}_{i}$ and ${Cls}_{i}$ denote the regression branch output and classification branch output for the *i*-th feature level, respectively. Critically, this output is modulated by a per-level, learnable scale factor, $a_{i}$, which is learned end-to-end. This mechanism enables effective scale-aware regression within a unified feature space, with the final bounding box coordinates obtained via the standard DFL decoding process [43].

## 4. Experiments

### 4.1. Experimental Setup

All experiments were conducted on a 64-bit Ubuntu 20.04 operating system, utilizing an AMD EPYC 7352 CPU (AMD, Santa Clara, CA, USA) and an NVIDIA GeForce RTX 4090 GPU (NVIDIA, Santa Clara, CA, USA). The models were implemented using PyTorch 2.3.0 (Meta AI, Menlo Park, CA, USA) with CUDA 12.4. (NVIDIA, Santa Clara, CA, USA) To ensure a fair comparison, all models were trained for 300 epochs with a batch size of 32. We used the SGD optimizer with a learning rate of 0.01, momentum of 0.937, and weight decay of 0.0005, following a cosine learning rate decay schedule. The key hyperparameters are summarized in Table 1.

YOLO-series baselines adopt the official open-source implementations released by the respective authors, while the remaining models are reproduced using the 3.3.0 MMDetection framework. Input resolutions follow the recommended settings of each framework: 640 × 640 for YOLO-series models and 1333 × 800 for MMDetection-based models (OpenMMLab, Shanghai, China). To ensure a fair comparison in terms of model complexity, all YOLO-series models use the medium-scale (‘m’) variant, and MSAdaNet adopts a matching channel configuration accordingly.

### 4.2. Datasets

#### 4.2.1. Augmented Smartphone Screen Glass Dataset

To validate the performance of our model on real-world data, we utilized the public SSGD. This dataset contains seven common defect types (Crack, Broken, Spot, Scratch, Light-Leakage, Blot, and Broken-Membrane) and is challenging due to numerous small, multi-category defects with complex shapes. To enhance robustness, we applied an offline augmentation pipeline that expanded the training set to five times its original size by generating four augmented versions per image. Each iteration randomly applied at least one of six operations: rotation within ±5° and scaling by a factor between 0.7 and 0.8; translation up to one-third of the image dimensions; brightness adjustment with a factor α ∈ [0.35, 1.0]; Gaussian noise injection; flipping horizontally, vertically, or diagonally with equal probability; and CutOut regularization using a 50 × 50 patch with an IoU threshold of 0.5 to preserve defect regions. Bounding box coordinates were updated for all geometric transformations. This expanded the original 1752 training images to 8760, as reported in Table 2. All compared models were trained on this identical augmented set to ensure fairness.

#### 4.2.2. Smartphone Camera Bezel Dataset

Due to the scarcity of defect data for camera bezels, a single defect-free sample image was captured as the base for data generation. To address data scarcity, we developed a synthetic pipeline to generate the Smartphone Camera Bezel Dataset (SCBD). The pipeline begins by transferring defect patterns from the NEU-DET dataset [44] to the base image, which matches color statistics in the LAB space to align defects with the target while preserving their structure [45]. It then applies Contrast Limited Adaptive Histogram Equalization (CLAHE) to the L-channel to enhance local contrast [46,47]. The process concludes with natural blending using Gaussian-smoothed alpha masks to avoid hard edges and ensure seamless integration [48]. This pipeline produced 4936 synthetic images containing six balanced defect classes, partitioned as detailed in Table 2.

### 4.3. Evaluation Metrics

We evaluated model performance using Precision (P), Recall (R), Average Precision (AP), and mean Average Precision (mAP) as the primary metric for detection accuracy. We report mAP@0.5 and mAP@0.5:0.95. Model complexity and speed were measured using Parameters (M), FLOPs (G), and inference speed in Frames Per Second (FPS). The core metrics of Precision (P), Recall (R), and Average Precision (AP) for a single class are defined as:

(20) $$Precision=\frac{TP}{TP+FP}\times 100\%$$

(21) $$Recall=\frac{TP}{TP+FN}\times 100\%$$

(22) $$AP=\int_{0}^{1}P(r)\;dr$$

(23) $$mAP=\frac{1}{m}\sum_{i=1}^{m}AP_{i}$$

where TP, FP, and FN represent true positives, false positives, and false negatives, respectively.

### 4.4. Results and Analysis

#### 4.4.1. Performance Comparison on the SSGD

To comprehensively evaluate MSAdaNet, we benchmarked it against eight state-of-the-art models (including Faster R-CNN, DETR [49], and YOLO variants) on the SSGD. As shown in Table 3, our proposed model demonstrates superior performance across key detection metrics.

MSAdaNet achieves the highest Recall of 59.5%, along with the best mAP@0.5 of 54.8% and mAP@0.5:0.95 of 26.9% among all evaluated models. Compared to the strong YOLOv11m baseline, our model shows a +18.3-point increase in Recall and a +10.6-point increase in mAP@0.5, with only a minor trade-off of −1.7 points in precision. This significant improvement in recall validates the effectiveness of our proposed modules in detecting difficult, fine-grained defects that are often missed by other models. It is worth noting that YOLOv12m yields a notably lower mAP@0.5 of 27.7% on the SSGD. A per-class analysis confirms that this degradation is consistent across all defect categories, which we attribute to the data-hungry nature of its attention-centric architecture struggling to converge effectively on this relatively small-scale industrial dataset.

Regarding computational complexity, MSAdaNet, with 28.4 M parameters and 113.2 GFLOPs, introduces a moderate overhead compared to YOLOv11m, which has 20.0 M parameters and 67.7 G FLOPs. It nevertheless maintains a clear efficiency advantage over two-stage detectors like Faster R-CNN, which requires 41.3 M parameters and 194 GFLOPs. We posit that for quality-critical inspection applications, the significant +10.6-point gain in mAP@0.5 justifies this well-managed trade-off between accuracy and efficiency.

#### 4.4.2. Performance Comparison on the SCBD

MSAdaNet achieves competitive performance on the SCBD, reaching 94.0% mAP@0.5. While Faster R-CNN achieves the highest precision, our method delivers superior results across all YOLO-series baselines, a full comparison of which is provided in Table 4. Specifically, MSAdaNet reaches 89.0% precision and 86.9% recall, improving recall by 5.4% over YOLOv11m. This enhancement in recall is especially critical for industrial quality control, where minimizing missed detections is essential to reduce costs. Against lightweight YOLO variants such as v10m, v11m, and v12m, MSAdaNet provides higher accuracy at a manageable computational cost of 28.4 million parameters and 113.2 GFLOPs. Furthermore, the lower result of DETR at 85.8% mAP indicates that CNN-based models are better suited to leveraging features from synthetic data for this particular application.

**Figure 7:**
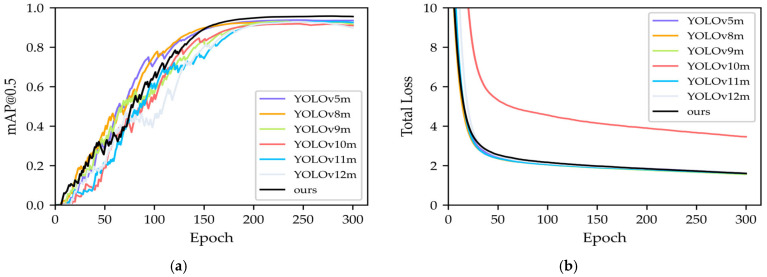
Training convergence on the SCBD: (**a**) mAP@0.5; (**b**) total loss.

Figure 7 shows that MSAdaNet converges stably and achieves the highest final metrics among YOLO variants, reaching a mAP@0.5 of 94.0% and a mAP@0.5:0.95 of 80.4%. This sustained mAP superiority during training validates the structural effectiveness of the proposed PMSFA, FDPN, and SASD modules for synthetic data.

### 4.5. Ablation Studies

We evaluated the contribution of each proposed module by adding components to the baseline YOLOv11m architecture. Experiments were conducted on both the SSGD and SCBD to assess the effectiveness and robustness of the architectural innovations across different data distributions.

#### 4.5.1. Ablation Study on the SSGD

Table 5 shows that each module contributes to the overall performance. Starting from a baseline mAP of 44.2% with 20.0 M parameters, PMSFA provides a significant improvement of 5.3% mAP. This suggests that parallel multi-scale feature extraction effectively captures defects across diverse scales despite an increase of 18.3 GFLOPs and a reduction in inference speed to 267.0 FPS. The addition of FDPN yields a further 1.7% mAP increase, which validates the focusing diffusion mechanism for multi-scale fusion. Finally, SASD contributes 3.6% mAP to reach a total of 54.8%, proving that scale adaptive transformations improve accuracy for extreme scale defects. The complete MSAdaNet achieves a 10.6% mAP gain over the baseline, justifying the computational overhead for quality critical smartphone defect inspection.

#### 4.5.2. Ablation Study on the SCBD

The ablation study on SCBD confirms consistent trends as shown in Table 6. PMSFA contributes 0.2% mAP while FDPN yields a decrease of 0.5% mAP, which likely stems from feature head mismatch on synthetic data. However, SASD delivers a 2.2% increase to reach 94.0% mAP. The consistent contributions across synthetic datasets validate the generalizability of the proposed design.

### 4.6. Visual Qualitative Analysis

**Figure 8:**
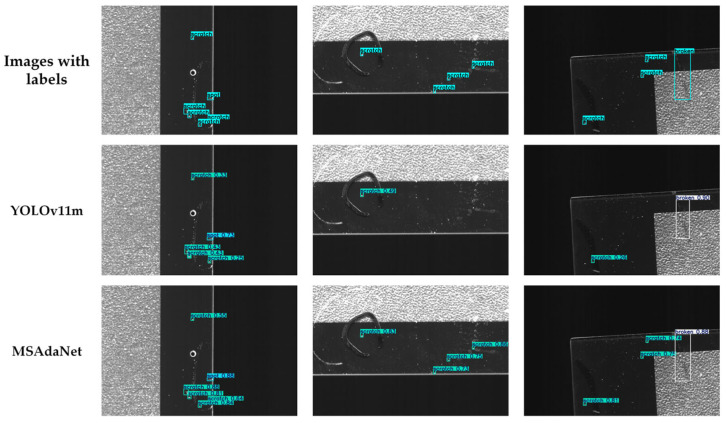
Comparative detection results of YOLOv11m and MSAdaNet on the SSGD.

**Figure 9:**
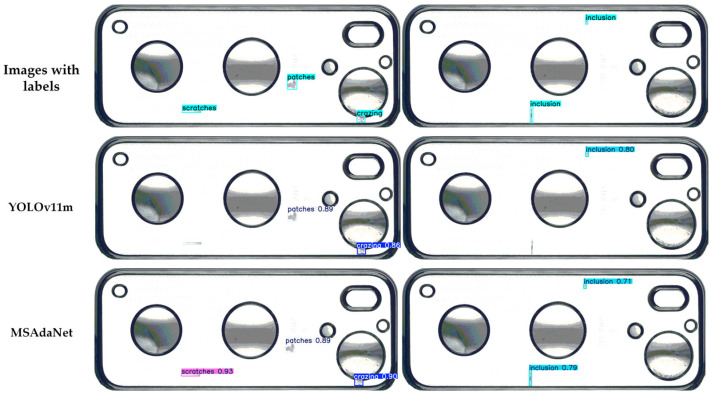
Comparative detection results of YOLOv11m and MSAdaNet on the SCBD.

Figure 8 compares the detection results and feature activation patterns of MSAdaNet against the baseline on the SSGD. The visual comparisons in Figure 8 and Figure 9 demonstrate that MSAdaNet excels in three areas. For small defects below 20 pixels, PMSFA captures fine-grained spatial details that the baseline misses. Due to the SASD module, MSAdaNet also demonstrates superior localization accuracy with bounding boxes that align closely with ground truth. In scenarios involving low contrast or textured surfaces, MSAdaNet maintains robust performance through the multi-scale contextual fusion of FDPN.

**Figure 10:**
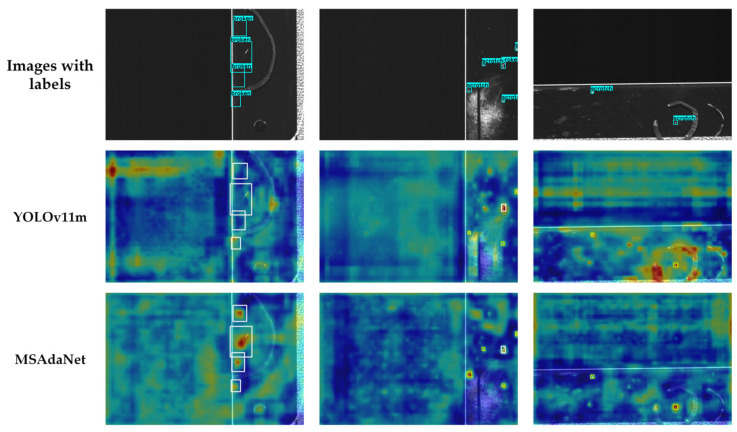
Comparative heatmaps of YOLOv11m and MSAdaNet for the SSGD.

**Figure 11:**
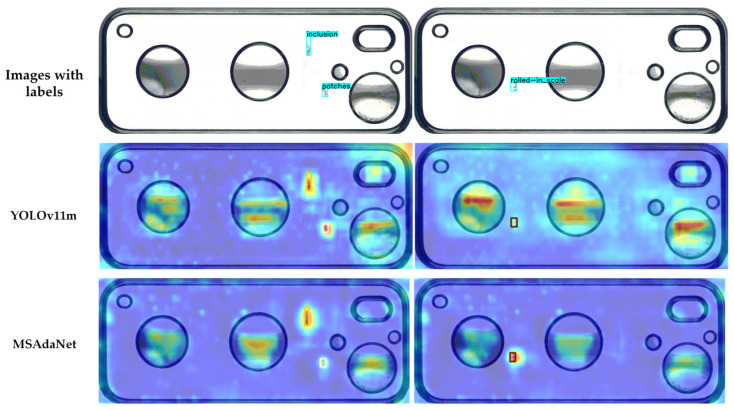
Comparative heatmaps of YOLOv11m and MSAdaNet for the SCBD.

Figure 10 and Figure 11 present Grad-CAM feature activation heatmaps where warmer colors indicate high activation intensities on defect regions [50]. While YOLOv11m exhibits diffuse attention patterns lacking sharp focus, MSAdaNet produces concentrated hotspots precisely aligned with actual defect locations. This focused pattern validates that the multi-receptive-field design extracts discriminative features effectively. MSAdaNet maintains strong activation even under weak visual signals, which demonstrates the capability of FDPN to amplify defect features through contextual fusion. The heatmaps also reveal that SASD maintains consistent attention quality across different scales regardless of object size.

These visual comparisons collectively demonstrate that MSAdaNet produces more precise bounding boxes and more concentrated feature activations than the baseline across both datasets. The Grad-CAM heatmaps confirm that the proposed modules enable the model to focus on defect-relevant areas rather than distributing attention diffusely, which is consistent with the quantitative improvements reported in the comparison and ablation experiments.

### 4.7. Discussions

#### 4.7.1. Advantages and Applications

Experiments and visualizations validate the superior detection performance of MSAdaNet on smartphone component defects. Leveraging high recall and multi-scale capabilities, it applies to screen glass inspection, camera bezel assessment, and downstream material screening. By integrating parallel multi-scale extraction, focusing diffusion fusion, and scale-adaptive regression, MSAdaNet enhances general detectors for industrial defect characteristics, with potential extension to other glass and optical surface inspection tasks.

#### 4.7.2. Limitations and Analyses

Although MSAdaNet has demonstrated strong detection performance, several limitations remain. Detection accuracy suffers for categories with insufficient labeled images. To mitigate data scarcity, we developed the SCBD as a cross-domain augmentation strategy. However, the quality of the synthetic data is currently validated only indirectly through downstream detection performance; more rigorous fidelity assessment using FID metrics and expert evaluation is required.

Computationally, MSAdaNet roughly doubles the complexity of YOLOv11m, it achieves 232.9 FPS on the SSGD and 375.1 FPS on the SCBD when tested on an RTX 4090, meeting server-side demands. However, these benchmarks may not translate to resource-constrained platforms like the NVIDIA Jetson series. On such devices, memory bandwidth limitations and thermal throttling can fundamentally alter the FLOPs-to-latency relationship, often making memory access patterns rather than computational throughput the primary bottleneck. Quantization (FP16/INT8) facilitates deployment, but the parallel convolutions of FDPN are unevenly sensitive to reduced precision, larger kernels in particular suffer greater quantization errors. Hence, thorough edge profiling and quantization analysis, along with edge-cloud collaboration, are essential to ensure real-world performance [51].

Per-class analysis reveals uneven performance. Blot and light-leakage defects suffer from high false positives, primarily because their low-contrast signatures blend with background textures, a phenomenon exacerbated by the multi-kernel enhancement of FDPN. Scratch-type defects, while effectively captured by the scale-adaptive regression of SASD, remain difficult to localize precisely under stricter IoU thresholds due to their extreme aspect ratios, which often deviate from typical anchor priors.

## 5. Conclusions

In this paper, we introduced MSAdaNet, a novel network designed for the surface defect detection of smartphone components. To address the fundamental challenges of extreme morphological and scale variation, we proposed three domain-specific modules: the PMSFA backbone for enhanced multi-scale feature capture, the application of a FDPN for efficient feature fusion, and a SASD head to improve localization accuracy. Furthermore, we developed a comprehensive data enrichment tool to combat data scarcity by generating a synthetic SCBD.

Extensive experiments validate our approach. On the real-world SSGD, MSAdaNet achieves a state-of-the-art mAP@0.5 of 54.8%, outperforming the YOLOv11m baseline by a substantial +10.6 points, driven largely by an +18.3-point gain in recall. On our synthetic SCBD, the model achieves 94.0% mAP@0.5, again surpassing all single-stage detectors and confirming the effectiveness of our data generation strategy. Ablation studies systematically confirmed that each proposed module contributes significantly to the final performance gains.

Despite these advances, certain limitations warrant further investigation, including dependence on labeled data quantity, the gap between server-grade and edge-device performance, and uneven detection accuracy across defect categories. Collecting more real-world defect images across various smartphone components, expanding the synthetic dataset with varied samples, and conducting fidelity validation using FID metrics and expert assessments would further strengthen the approach. Developing lightweight MSAdaNet variants through pruning and knowledge distillation, coupled with latency benchmarking on edge platforms and robustness evaluation under quantization, represents a step toward practical embedded deployment. Evaluation on additional public industrial defect benchmarks would also help validate generalization, while aspect-ratio-aware regression strategies and weakly supervised detection methods offer directions for improving performance under challenging defect geometries and data-scarce conditions.

## Figures and Tables

**Table 1:** Experimental parameters.

Parameter	Value
Learning rate	0.01
Decay strategy	cosine
Optimizer	SGD
Momentum	0.937
Weight decay	0.0005
Total epochs	300
Close mosaic	0
Batch size	32

**Table 2:** SSGD and SCBD.

Dataset	Total Images	Defect Classes	Train	Validation	Test
SSGD	9512	7	8760	251	501
SCBD	4936	6	3455	494	987

**Table 3:** Results on the SSGD dataset of different models.

Model	Precision (%)	Recall (%)	mAP@0.5 (%)	mAP@0.5:0.95 (%)	Parameters (M)	FLOPs (G)
Faster R-CNN	51.2	53.4	52.3	22.5	41.3	194
DETR	44.7	52.6	42.8	18.5	41.5	225
YOLOv5m	55.4	43.0	45.6	21.3	25.0	64.0
YOLOv8m	47.5	50.7	47.2	21.7	25.8	78.7
YOLOv9m	45.0	51.9	49.5	22.6	20.0	76.5
YOLOv10m	**61.4**	42.7	42.4	20.2	**15.3**	**58.9**
YOLOv11m	58.6	41.2	44.2	19.6	20.0	67.7
YOLOv12m	29.1	30.3	27.7	11.0	20.1	67.1
Ours	56.9	**59.5**	**54.8**	**26.9**	28.4	113.2

**Table 4:** Results on the SCBD dataset of different models.

Model	Precision (%)	Recall (%)	mAP@0.5 (%)	mAP@0.5:0.95 (%)	Parameters (M)	FLOPs (G)
Faster R-CNN	**97.3**	**92.7**	**97.8**	**80.6**	41.3	194
DETR	82.9	81.7	85.8	55.9	41.5	225
YOLOv5m	87.4	83.4	92.1	78.3	25.0	64.0
YOLOv8m	86.8	82.6	91.5	77.7	25.8	78.7
YOLOv9m	88.4	83.2	91.9	78.7	20.0	76.5
YOLOv10m	88.1	80.6	91.3	77.3	**15.3**	**58.9**
YOLOv11m	87.2	81.5	92.1	78.3	20.0	67.7
YOLOv12m	86.8	79.8	91.6	77.0	20.1	67.1
Ours	89.0	86.9	94.0	80.4	28.4	113.2

**Table 5:** Ablation study results on the SSGD.

Methods	mAP@0.5	mAP@0.5:0.95 (%)	Parameters (M)	FLOPs (G)	FPS
Baseline (YOLOv11m)	44.2	19.6	**20.0**	**67.7**	**388.8**
+PMSFA	49.5 (+5.3)	23.5 (+3.9)	20.6 (+0.6)	86.0 (+18.3)	267.0 (−121.8)
+PMSFA, FDPN	51.2 (+1.7)	25.1 (+1.6)	25.9 (+5.3)	102.8 (+16.8)	223.6 (−43.4)
+PMSFA, FDPN, SASD	**54.8 (+** **3.6** **)**	**26.9** **(+1.8)**	28.4 (+2.5)	113.2 (+10.4)	232.9 (+9.3)

**Table 6:** Ablation study results on the SCBD.

Methods	mAP@0.5	mAP@0.5:0.95 (%)	Parameters (M)	FLOPs (G)	FPS
Baseline (YOLOv11m)	92.1	78.3	**20.0**	**67.7**	**593.7**
+PMSFA	92.3 (+0.2)	77.8 (−0.5)	20.6 (+0.6)	86.0 (+18.3)	535.2 (−58.5)
+PMSFA, FDPN	91.8 (−0.5)	78.9 (+1.1)	25.9 (+5.3)	102.8 (+16.8)	366.7 (−168.5)
+PMSFA, FDPN, SASD	**94.0** **(+2.2)**	**80.4** **(+1.5)**	28.4 (+2.5)	113.2 (+10.4)	375.1 (+8.4)

## Data Availability

The SSGD presented in the study is openly available on GitHub at https://github.com/VincentHancoder/SSGD, accessed on 20 June 2025.
